# PD-1/PD-L1 expression in anal squamous intraepithelial lesions

**DOI:** 10.18632/oncotarget.27756

**Published:** 2020-09-29

**Authors:** Margot Bucau, Nathalie Gault, Nanthara Sritharan, Emy Valette, Charlotte Charpentier, Francine Walker, Anne Couvelard, Laurent Abramowitz

**Affiliations:** ^1^AP-HP, Département de Pathologie, Hôpital Bichat-Claude Bernard, F-75018 Paris, France; ^2^AP-HP, Département d’Epidémiologie Biostatistiques et Recherche Clinique, Hôpital Bichat-Claude Bernard, F-75018 Paris, France; ^3^INSERM CIC-EC1425, Hôpital Bichat-Claude Bernard, F-75018 Paris, France; ^4^AP-HP, Service de Gastroentérologie et Proctologie, Hôpital Bichat-Claude Bernard, F-75018 Paris, France; ^5^AP-HP, Laboratoire de Virologie, Hôpital Bichat-Claude Bernard, F-75018 Paris, France; ^6^Université de Paris, INSERM UMR 1137 IAME, F-75018 Paris, France; ^7^Université de Paris, INSERM UMR 1149, F-75018 Paris, France; ^8^Ramsay GDS, Clinique Blomet, 75015 Paris, France

**Keywords:** anal dysplasia, PD-L1, HPV, anal cancer, immune microenvironment

## Abstract

Introduction: Studies have shown that the PD-1/PD-L1 immunomodulatory pathway slows down anti-tumor immunity in a number of cancers. The description of the expression of these molecules has never been performed in anal low-grade/high grade squamous intra-epithelial lesions (LSIL/HSIL respectively).

Materials and Methods: Patients followed in the AIN3 cohort were routinely sampled. For each selected sample, an immunohistochemical study was performed with anti-CD8, PD-1, PD-L1 antibodies. The presence and distribution of CD8+ lymphocytes, and the presence of PD-1+ lymphocytes and PD-L1+ epithelial cells were assessed. The comparison of these characteristics was performed between the HSIL and LSIL groups.

Results: 33 patients were included and 78 samples selected (60 HSIL and 18 LSIL). CD8+ lymphocytes were observed more frequently in HSIL versus LSIL in the lamina propria or intra epithelial (respectively 90% vs. 60%, *p* = 0.01; and 62% vs. 33%, *p* = 0.04). PD-1+ lymphocytes were observed more frequently in HSIL versus LSIL (41% vs 11%, *p* = 0.03). There was no difference between HSIL and LSIL for PD-L1+ epithelial cells.

Conclusions: Anal dysplastic lesions are accompanied by an inflammatory lymphocytic infiltrate expressing CD8 and PD-1, more frequent in high-grade lesions. These results highlight the involvement of the PD-1/PD-L1 pathway in the natural history of anal dysplasia.

## INTRODUCTION

Anal intraepithelial neoplasia (AIN) is the precursor lesion for anal squamous cell carcinomas (ASCC). GLOBOCAN survey estimated 40 000 new cases of ASCC worldwide in 2012, 88% of which due to persistent HPV infection [1, 2]. Risk factors for developing AIN and/or ASCC are sexual behaviors (especially men who have sex with men), women with previous human papillomavirus (HPV)-related disease, HIV infection, and chronic intake of immunosuppressant.

Since 2012, the Lower Anogenital Squamous Terminology (LAST) [3] recommended denomination for HPV-associated squamous lesions of the lower anogenital tract as low-grade and high-grade squamous intraepithelial lesion (LSIL and HSIL respectively). They may be further classified by the applicable Anal Intraepithelial Neoplasia (AIN) categorization into mild dysplasia AIN1 (LSIL), moderate dysplasia AIN2 and severe dysplasia AIN3 (both of them HSIL).

Although much less studied than cervical cancer and precursors, AIN and ASCC may have quite similar progression model, however progression rate to cancer is lower [4]. Oncogenic HPV infection plays a crucial role in developing both cervical and anal lesions, by integration of the viral DNA into the epithelial cells and activation of oncogenic early proteins E6 and E7. This causes downregulation of suppressing tumors genes, especially TP53 and Rb, and upregulation of p16 [5]. In the cervix, HPV related cancer often have increased infiltration by immune cell populations, including cytotoxic CD8+ T cells, that correlates with better response to chemo radiotherapy and increased survival compared to immune-deprived tumors. Moreover, p16 positive tumors were shown to present higher tumor infiltrating lymphocytes density and better recurrence-free survival [6]. Interestingly, PD-1/PD-L1 inhibitors have shown efficacy in treating cervical cancer [7]. In this context, some authors evaluated PD-L1 expression in ASCC, with conflicting results. In a large series of 150 patients, Blermapas and al [8]. described that a high amount of PD-1 positive tumor infiltrating lymphocytes was associated with better disease-free survival. In contrast, others found that PD-L1 expression was associated with a worse prognosis [9, 10]. Immune checkpoint inhibition has only been reported in a limited series of patients with locally advanced, recurrent or metastatic ASCC, showing 24% of tumor response [11]. To our knowledge, no study has investigated the PD-1/PD-L1 pathway in pre-cancerous anal lesions, whereas scarce data exist in cervical intra-epithelial neoplasia [12–15]. The aim of our study was to explore PD-1/PD-L1 pathway and immune infiltration in a prospective cohort of patients presenting anal intraepithelial neoplasia of different grades.

## RESULTS

We included 78 samples of 33 patients (60 AIN2/AIN3 and 18 AIN1). Among 18 LSIL/AIN1, we found 7 condylomas with no apparent dysplasia and 11 flat AIN1 lesions. Patients were mainly male (*n* = 23, 69.7%), HIV-positive (*n* = 24, 72.7%), with a median age of 49 years (interquartile range 43–59).

Six patients had only one high grade available sample, 9 patients had two high grade available samples, and 18 had two high grade available samples plus one low grade sample. In total, we had at our disposal 78 samples: 18 low grade lesions (AIN1), 7 high grade lesions (AIN2) and 53 high grade lesions (AIN3). Among all these samples, 60 were biopsies and 18 surgical excision specimens.

**Figure 1:**
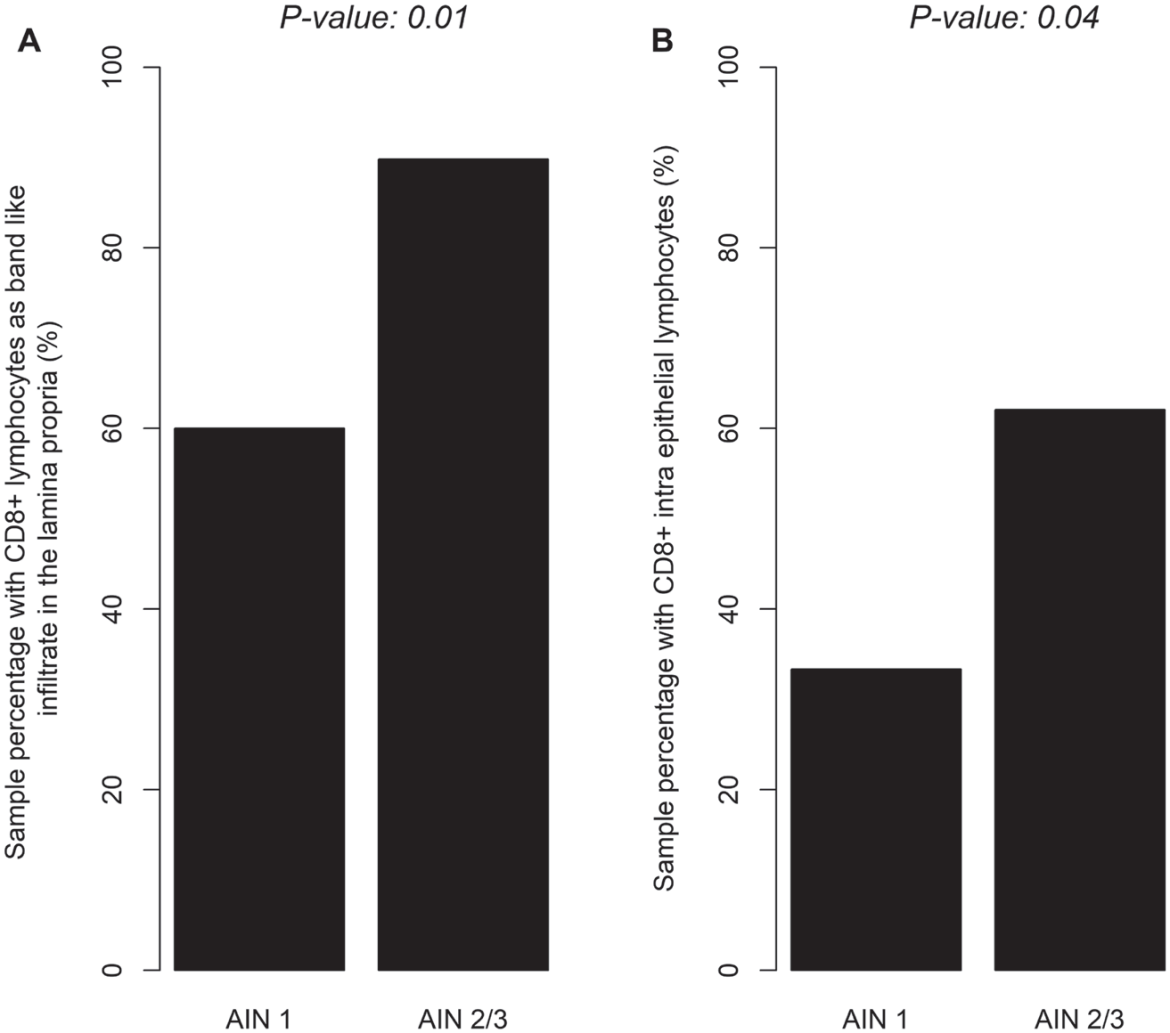
Distribution of CD8+ band like lymphocytic infiltrate in lamina proria (**A**) or CD8+ intra epithelial lymphocytes (**B**) according to severity of dysplasia (LSIL/HSIL).

CD8+ lymphocytes were distributed as band like infiltrate in the lamina propria. Fourteen samples were not analyzable as a result of material loss, 11 had no band-like infiltrate (score 0), 22 had sporadic CD8 lymphocytes (score 1), 17 had a moderate amount of CD8+ lymphocytes (score 2), 14 had an abundant amount of CD8+ band-like lymphocytes infiltrate (score 3). In summary, 53/78 lesions had a CD8+ band-like infiltrate (68%). Inside the epithelium, there were 42/78 samples with CD8+ lymphocytes (54%). Forty-four (90%) AIN2/AIN3 samples had CD8+ lymphocytes in the lamina propria as compared to 9 (60%) in AIN1 samples (*p* = 0.01) (Figure 1A). Similarly, 36 (62%) AIN2/AIN3 samples had CD8+ intra epithelial lymphocytes versus 6 (33%) AIN1 samples (*p* = 0.04) (Figure 1B).

**Figure 2:**
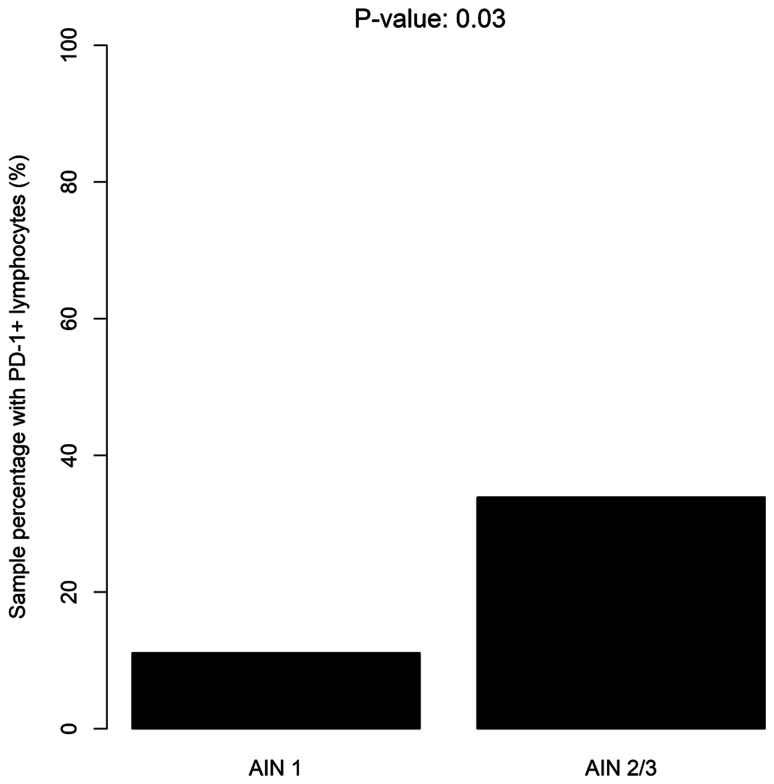
Distribution of PD-1+ lymphocytes according to the severity of dysplasia (LSIL/HSIL).

PD-1+ lymphocytes were detected in band-like infiltrate in the lamina propria. One sample was not analyzable as a result of material loss, 55 had no PD-1+ lymphocytes in the lamina propria (score 0), 12 had sporadic PD-1+ lymphocytes (score 1), 6 had a moderate amount of PD-1+ lymphocytes (score 2), 4 had an abundant amount of PD-1+ lymphocytes infiltrate (score 3). In summary, 22/78 lesions had a PD-1+ band-like infiltrate (28%). We found no intra epithelial PD-1+ lymphocytes. Twenty-four (41%) AIN2/AIN3 samples contained PD-1+ lymphocytes as compared to 2 (11%) AIN1 samples (*p* = 0.03) (Figure 2).

**Figure 3:**
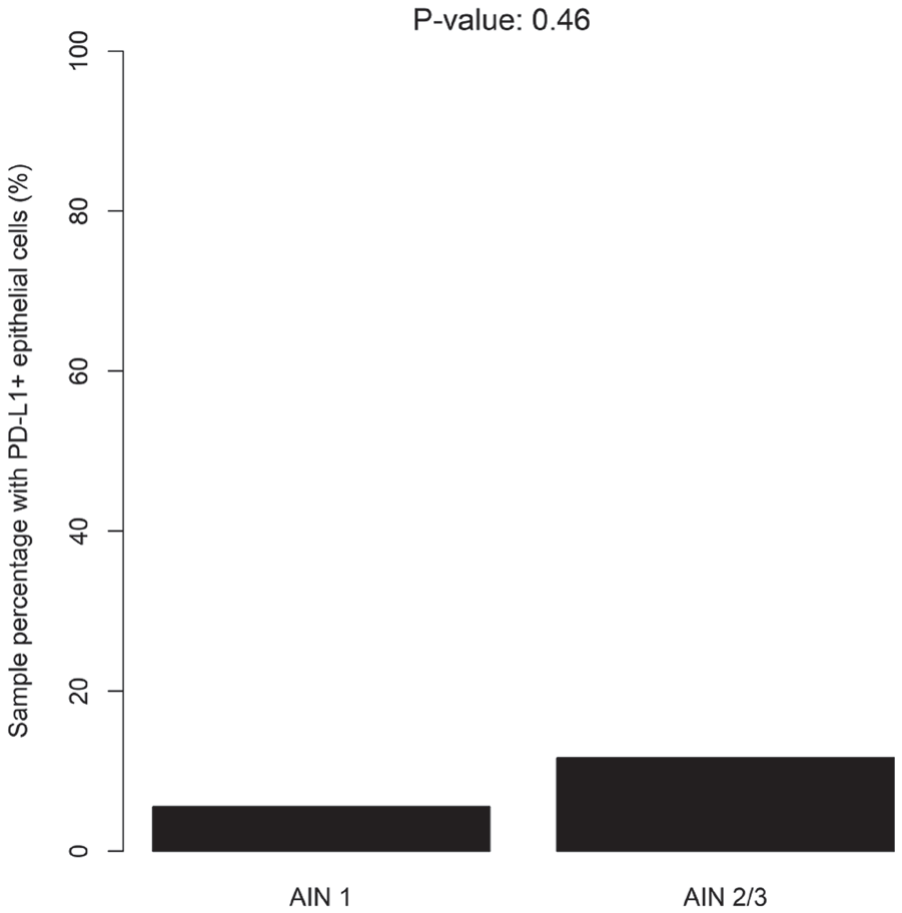
Distribution of PD-L1+ epithelial cells according to the severity of dysplasia (LSIL/HSIL).

The rate of PD-L1 marked epithelial cells was comparable between different grades of dysplasia: there was one (6%) AIN1 sample with PD-L1+ epithelial cells and 7 (12%) AIN2/3 samples (*p* = 0.43; Figure 3). The distribution of PD-L1+ lymphocytes was comparable between AIN2/AIN3 and AIN1 samples: 3 (16%) versus 15 (25%), respectively; *p* = 0.47.

Since we had only one AIN1 lesion in HIV negative patients, we did not compare AIN1 lesion between the two groups. We analyzed our markers in the AIN2/AIN3 group containing 14 HIV- patients and 46 HIV+ patients: 7 patients (70%) had CD8+ lymphocytes in the lamina propria in the HIV negative group as compared to 37 (95%) in the HIV positive group (*p* = 0.05). There was no difference between the two groups for the others markers.

**Figure 4:**
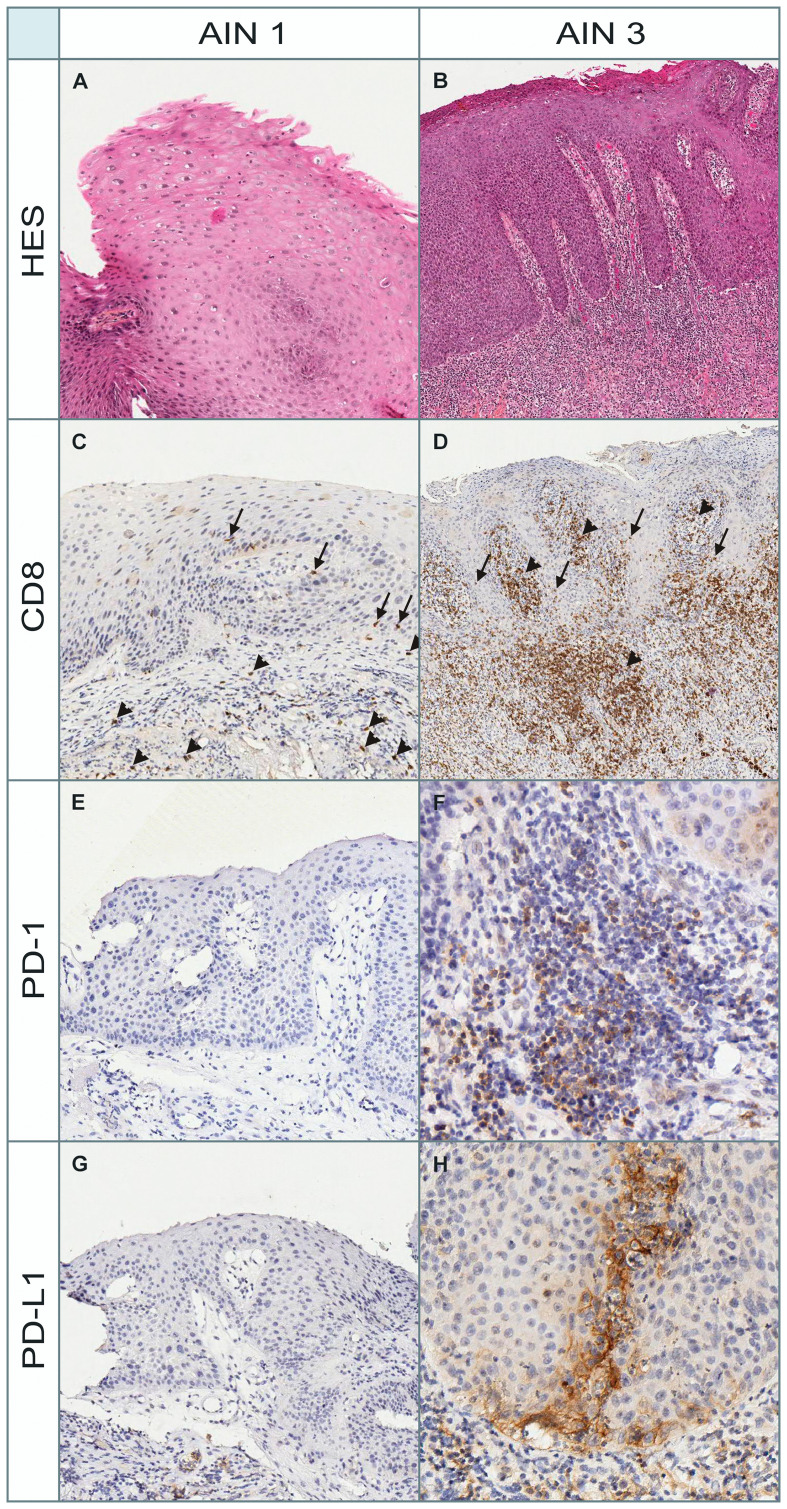
Morphological and immunohistochemical (PD-1, PD-L1, and CD8) aspects in a patient with AIN1 and AIN3. HES: Hematoxylin, Eosin and Saffron stain for morphological assessment. (**A**) AIN1 lesion stained with HES. (**B**) AIN3 lesion stained with HES. (**C**) CD8 immunostain scored 1 for both band-like infiltrate (arrowheads) and intra-epithelial lymphocytes (arrows) in AIN1 lesion. (**D**) CD8 immunostain scored 3 and 1 respectively for band-like infiltrate (arrowheads) and intra-epithelial lymphocytes (arrows) in AIN3 lesion. (**E**) PD-1 immunostain (score 0: no infiltrate) in AIN1 lesion. (**F**) PD-1 immunostain (score 3: abondant lymphocytic infiltrate) in AIN3 lesion. (**G**) PD-L1 immunostain (no positive epithelial cells or lymphocytes) in AIN1 lesion. (**H**) PD-L1 immunostain (40% of PD-L1+ epithelial cells) in AIN3 lesion. The positive cells are large epithelial squamous cells and no lymphocytes are marked.

Figure 4 illustrates different immunostainings (PD-1, PD-L1, and CD8) and the standard morphology on HES (Hematoxylin, Eosin and Saffron stain) of one patient with AIN1 and AIN3. The AIN1 lesion (A), shows (C) few band-like infiltrate or intra-epithelial CD8+ lymphocytes (both of them scored 1), (E) no PD-1+ lymphocytes, (G) and no PD-L1+ epithelial cells. In contrast, the AIN3 lesion (B), shows (D) an abundant band-like infiltrate and intra-epithelial CD8+ lymphocytes (scored 3 and 1 respectively), (F) a high number of PD-1+ lymphocytes (score 3), and (H) 40% of PD-L1+ epithelial cells, mainly located close to the basal membrane.

## DISCUSSION

To our knowledge, this is the first study analyzing the PD-1 pathway in anal squamous intra epithelial lesions.

We found an increase of CD8+ lymphocytes infiltration, both in the lamina propria and within the epithelium, in high-grade lesions. Recruitment of lymphocytes CD8 in dysplasia progression has been described in cervical lesions [16]. This recruitment may have a role in viral clearance in regressing cervical intra epithelial lesion [17]. Furthermore, a decreased number of CD8+ lymphocytes and their association with PD-L1 expression in tumor cells has also been described in anal squamous cell carcinoma [9], suggesting that activation of PD-L1 pathway may induce a immune tolerant profile accelerating immune escapement of tumor cells.

In our study, HSIL had more PD-1+ lymphocytes as compared to LSIL. This result is consistent with most studies on PD-1/PD-L1 axis in cancers. PD-1 is a transmembrane receptor expressed by activated T cells and B cells. Activation of the PD-1/PD-L1 pathway leads to tumor-infiltrating lymphocytes (TILs) dysfunction in several cancers [18]. Dependence between CD8+ intra-epithelial lymphocytes and PD-1+ lymphocytes supports the idea of the crucial role of cytotoxic cells in the neoplastic progression. In this respect, Mezache et al. [12] found in co-expression analysis that the majority of the PD-L1+ mononuclear stromal cells were CD8+ cells. We found no association between CD8+ band-like infiltrate in the lamina propria and PD-1/PD-L1+ lymphocytes. This result suggests a direct role of HPV within the epithelium, activating PD-1/PD-L1 pathway and therefore stopping the anti tumoral activity of cytotoxic lymphocytes. These findings also support the use of Imiquimod in anal dysplasia [19]. Imiquimod induces high levels of interferon-alpha (IFN-*α*), tumor necrosis factor alpha (TNF-*α*), and other interleukins, activating innate immune system and potentially activating cytotoxic T lymphocytes and NK cells [20]. Furthermore, Ooi et al. [21] showed statistically significant increases of CD3, CD4, CD8, CD11c, CD86/CD11c lymphocytes, and CD68 macrophages positive cells by immunohistochemistry after 2 weeks of Imiquimod treatment in a series of 18 patients with skin actinic keratosis.

Interestingly, we found little number of dysplastic lesions with PD-L1+ epithelial cells. Nearly all positive cases were high grade lesions. This is somewhat unusual in regards to most data on cervical intra epithelial neoplasia showing an increasing number of PD-L1+ epithelial cells with dysplasia progression in most samples. For instance, Mezache et al. [12] found 20/21 samples with at least 10% PD-L1+ epithelial cell in their CIN1-2 cohort group, and Yang et al. [15] found 8 out of 10 sample positive for PD-L1. Only one study conducted by Chang et al. [22] found no positive staining for PD-L1 in epithelial cell. In the case anal dysplasia, this could be explained by the small analyzed area where the majority of our samples consist of small biopsies. A recent study reported 56% of PD-L1 positive anal cancer samples [10], and positive lesions had a less favorable prognosis (increased local recurrence and mortality rates) supporting the hypothesis that anti PD-1/PD-L1 therapy may be effective to treat this invasive neoplasia. Eventually, a phase-Ib study published in 2017 [23] tested pembrolizumab in patients with advanced anal carcinoma: out of 43 patients, 32 (74%) had PD-L1 epithelial expression and resulting in 58% of disease control rate.

Regarding difference between HIV positive and HIV negative patients, we found a greater amount of CD8+ lymphocytes in the lamina propria of the AIN2/AIN3 lesions in the HIV positive group. This result could be related with the antiviral activities of CD8+ lymphocytes during HIV infection [24].

Our study has limitations. First, despite the scale of our series (greatest AIN3 series in European countries), we only had access to a limited number of samples, because anal dysplasia is much rarer than cervical dysplasia with a long dysplastic progression. Patient’s samples were often small or already spent for the diagnosis, leading to a difficult analysis of the immune microenvironment in some cases. We did not find a lot of low grade lesions since they are infrequently biopsied or excised by physicians. The study the natural history of dysplasia progression is very challenging since most patients developed several lesions, some of these spontaneously regressing, but not some of them.

In summary, our exploratory study highlights the interest of the PD-1/PD-L1 pathway in anal dysplasia and the importance to further explore the different mechanisms of immune micro environment in the progression of anal intra epithelial lesion. It suggests the potential role of therapeutic molecules targeting the immune response to slow down the tumor progression in selected patients with HSIL.

## MATERIALS AND METHODS

We conducted a case-control study nested in the AIN3 cohort (NCT01877135). Briefly, this French multicenter cohort aims at studying the incidence of anal epidermoid carcinoma in the 3 years following a first diagnosis of severe dysplasia (AIN3). Adult patients are included at the date of first diagnosis of AIN3. Follow-up includes clinical examination with an anal smear sample at least once a year, and anal biopsies if appropriate. All patients gave their informed consent to the study.

Eligible patients were those included in the AIN3 cohort and having at least one biopsy sample stored in the Department of Pathology of Bichat hospital since the year 2000. All available pathological specimens were retrieved. All the slides were reviewed by two pathologists (AC and MB). Diagnosis of AIN was made according to the Lower Ano-genital Squamous Terminology [3]: LSIL and HSIL with further qualification in mild (AIN1/LSIL), moderate (AIN2/HSIL) and severe (AIN3/HSIL). For patients with several samples, we selected the first available AIN3 sample and, if available, another high grade lesion (AIN2 or AIN3). If disposable in our laboratory, a low grade lesion (AIN1) was also selected for each patient.

Immunohistochemistry was performed with an automated immunohistochemical stainer according to the manufacturer’s guidelines (Streptavidine-peroxidase with an automate Leica Bond III, USA). Four μm sections were dewaxed, rehydrated and antigen retrieval was conducted by pretreatment at high temperature at pH9 in TRIS buffer, during 30 minutes. Slides were immunolabelled with monoclonal antibodies against CD8 (1:300, C8/144B; Dako), PD-1 (1:100, AB52587, Abcam) and PD-L1 (1:00, 13684 Cell Signaling). The estimation of the staining was performed on the whole tissue section for each sample.

We scored the lymphocytes expressing PD-1, PD-L1 or CD8 and their location (intra-epithelial and/or intra-lamina propria) and the epithelial cells expressing PD-L1.

(A) In the lamina propria forming band-like infiltrate, the expression of the markers on lymphocytes was scored semi-quantitatively as follows: (0) absence of infiltrate [1], sporadic discontinuous band-like lymphocytic infiltrate [2]; band-like lymphocytic infiltrate of moderate importance [3]; abundant band like lymphocytic infiltrate.

(B) In the epithelium, the expression of the markers on intraepithelial lymphocytes was scored semi-quantitatively as follows: (0) absence of intra-epithelial lymphocytes [1], sporadic lymphocytes [2]; moderate numbers of lymphocytes [3]; abundant lymphocytes.

We further classified band-like infiltrate and intra-epithelial lymphocytes as present (score ≥ 1) or not (score 0).

(C) We quantified the percentage of stained epithelial cells expressing of PD-L1. We further considered the epithelium as positive for PD-L1 when the percentage exceeded 5%.

These data were compared between case (high grade intra-epithelial lesions) and control (low grade intra-epithelial lesions) samples by the mean of a Wald test on a mixed model taking into account multiple samples per patient. We further analyzed difference between HIV positive and HIV negative patients in the AIN2/AIN3 group.

All tests were 2-sided and significance level was set at 5%.

## References

[1] Schiffman M , Doorbar J , Wentzensen N , de Sanjosé S , Fakhry C , Monk BJ , Stanley MA , Franceschi S . Carcinogenic human papillomavirus infection. Nat Rev Dis Primers. 2016; 2:16086. 10.1038/nrdp.2016.86. 27905473

[2] De Vuyst H , Clifford GM , Nascimento MC , Madeleine MM , Franceschi S . Prevalence and type distribution of human papillomavirus in carcinoma and intraepithelial neoplasia of the vulva, vagina and anus: A meta-analysis. Int J Cancer. 2009; 124:1626–1636. 10.1002/ijc.24116. 19115209

[3] Darragh TM , Colgan TJ , Cox JT , Heller DS , Henry MR , Luff RD , McCalmont T , Nayar R , Palefsky JM , Stoler MH , Wilkinson EJ , Zaino RJ , Wilbur DC , et al. The Lower Anogenital Squamous Terminology Standardization Project for HPV-Associated Lesions: Background and Consensus Recommendations from the College of American Pathologists and the American Society for Colposcopy and Cervical Pathology. Arch Pathol Lab Med. 2012; 136:1266–1297. 10.5858/arpa.LGT200570. 22742517

[4] Machalek DA , Poynten M , Jin F , Fairley CK , Farnsworth A , Garland SM , Hillman RJ , Petoumenos K , Roberts J , Tabrizi SN , Templeton DJ , Grulich AE . Anal human papillomavirus infection and associated neoplastic lesions in men who have sex with men: a systematic review and meta-analysis. Lancet Oncol. 2012; 13:487–500. 10.1016/S1470-2045(12)70080-3. 22445259

[5] Martin D , Rödel F , Balermpas P , Rödel C , Fokas E . The immune microenvironment and HPV in anal cancer: Rationale to complement chemoradiation with immunotherapy. Biochim Biophys Acta Rev Cancer. 2017; 1868:221–230. 10.1016/j.bbcan.2017.05.001. 28501560

[6] Gilbert DC , Serup-Hansen E , Linnemann D , Høgdall E , Bailey C , Summers J , Havsteen H , Thomas GJ . Tumour-infiltrating lymphocyte scores effectively stratify outcomes over and above p16 post chemo-radiotherapy in anal cancer. Br J Cancer. 2016; 114:134–137. 10.1038/bjc.2015.448. 26730577 PMC4815814

[7] Frenel JS , Le Tourneau C , O’Neil B , Ott PA , Piha-Paul SA , Gomez-Roca C , van Brummelen EMJ , Rugo HS , Thomas S , Saraf S , Rangwala R , Varga A . Safety and Efficacy of Pembrolizumab in Advanced, Programmed Death Ligand 1-Positive Cervical Cancer: Results From the Phase Ib KEYNOTE-028 Trial. J Clin Oncol. 2017; 35:4035–4041. 10.1200/JCO.2017.74.5471. 29095678

[8] Balermpas P , Martin D , Wieland U , Rave-Fränk M , Strebhardt K , Rödel C , Fokas E , Rödel F . Human papilloma virus load and PD-1/PD-L1, CD8 + and FOXP3 in anal cancer patients treated with chemoradiotherapy: Rationale for immunotherapy. Oncoimmunology. 2017; 6:e1288331. 10.1080/2162402X.2017.1288331. 28405521 PMC5384387

[9] Zhao YJ , Sun W , Peng JH , Deng YX , Fang Y , Huang J , Zhang H , Wan D , Lin J , Pan ZZ . Programmed death-ligand 1 expression correlates with diminished CD8+ T cell infiltration and predicts poor prognosis in anal squamous cell carcinoma patients. Cancer Manag Res. 2017; 10:1–11. 10.2147/CMAR.S153965. 29296096 PMC5739110

[10] Govindarajan R , Gujja S , Siegel ER , Batra A , Saeed A , Lai K , James JD , Fogel BJ , Williamson S . Programmed Cell Death-Ligand 1 (PD-L1) Expression in Anal Cancer. Am J Clin Oncol. 2018; 41:638–642. 10.1097/COC.0000000000000343. 27849650

[11] Morris VK , Salem ME , Nimeiri H , Iqbal S , Singh P , Ciombor K , Polite B , Deming D , Chan E , Wade JL , Xiao L , Bekaii-Saab T , Vence L , et al. Nivolumab for previously treated unresectable metastatic anal cancer (NCI9673): a multicentre, single-arm, phase 2 study. Lancet Oncol. 2017; 18:446–453. 10.1016/S1470-2045(17)30104-3. 28223062 PMC5809128

[12] Mezache L , Paniccia B , Nyinawabera A , Nuovo GJ . Enhanced expression of PD L1 in cervical intraepithelial neoplasia and cervical cancers. Mod Pathol. 2015; 28:1594–1602. 10.1038/modpathol.2015.108. 26403783

[13] Yang-Chun F , Zhen-Zhen C , Yan-Chun H , Xiu-Min M . Association between PD-L1 and HPV status and the prognostic value for HPV treatment in premalignant cervical lesion patients. Medicine. 2017; 96:e7270. 10.1097/MD.0000000000007270. 28640134 PMC5484242

[14] Yang W , Song Y , Lu YL , Sun JZ , Wang HW . Increased expression of programmed death (PD)-1 and its ligand PD-L1 correlates with impaired cell-mediated immunity in high-risk human papillomavirus-related cervical intraepithelial neoplasia. Immunology. 2013; 139:513–522. 10.1111/imm.12101. 23521696 PMC3719068

[15] Yang W , Lu YP , Yang YZ , Kang JR , Jin YD , Wang HW . Expressions of programmed death (PD)-1 and PD-1 ligand (PD-L1) in cervical intraepithelial neoplasia and cervical squamous cell carcinomas are of prognostic value and associated with human papillomavirus status: PD-1/PD-L1 in CIN and cervical carcinomas. J Obstet Gynaecol Res. 2017; 43:1602–1612. 10.1111/jog.13411. 28833798

[16] Monnier-Benoit S , Mauny F , Riethmuller D , Guerrini JS , Căpîlna M , Félix S , Seillès E , Mougin C , Prétet JL . Immunohistochemical analysis of CD4+ and CD8+ T-cell subsets in high risk human papillomavirus-associated pre-malignant and malignant lesions of the uterine cervix. Gynecol Oncol. 2006; 102:22–31. 10.1016/j.ygyno.2005.11.039. 16427684

[17] Trimble CL , Clark RA , Thoburn C , Hanson NC , Tassello J , Frosina D , Kos F , Teague J , Jiang Y , Barat NC , Jungbluth AA . Human Papillomavirus 16-Associated Cervical Intraepithelial Neoplasia in Humans Excludes CD8 T Cells from Dysplastic Epithelium. J Immunol. 2010; 185:7107–14. 10.4049/jimmunol.1002756. 21037100 PMC3075978

[18] Dong P , Xiong Y , Yue J , Hanley SJB , Watari H . Tumor-Intrinsic PD-L1 Signaling in Cancer Initiation, Development and Treatment: Beyond Immune Evasion. Front Oncol. 2018; 8:386. 10.3389/fonc.2018.00386. 30283733 PMC6156376

[19] Richel O , de Vries HJ , van Noesel CJ , Dijkgraaf MG , Prins JM . Comparison of imiquimod, topical fluorouracil, and electrocautery for the treatment of anal intraepithelial neoplasia in HIV-positive men who have sex with men: an open-label, randomised controlled trial. Lancet Oncol. 2013; 14:346–353. 10.1016/S1470-2045(13)70067-6. 23499546

[20] Voiculescu VM , Lisievici CV , Lupu M , Vajaitu C , Draghici CC , Popa AV , Solomon I , Sebe TI , Constantin MM , Caruntu C . Mediators of Inflammation in Topical Therapy of Skin Cancers. Mediators Inflamm. 2019; 2019:8369690. 10.1155/2019/8369690. 30766448 PMC6350587

[21] Ooi T , Barnetson RS , Zhuang L , McKane S , Lee JH , Slade HB , Halliday GM . Imiquimod-induced regression of actinic keratosis is associated with infiltration by T lymphocytes and dendritic cells: a randomized controlled trial. Br J Dermatol. 2006; 154:72–78. 10.1111/j.1365-2133.2005.06932.x. 16403097

[22] Chang H , Hong JH , Lee JK , Cho HW , Ouh YT , Min KJ , So KA . Programmed death-1 (PD-1) expression in cervical intraepithelial neoplasia and its relationship with recurrence after conization. J Gynecol Oncol. 2018; 29:e27. 10.3802/jgo.2018.29.e27. 29400020 PMC5920214

[23] Ott PA , Piha-Paul SA , Munster P , Pishvaian MJ , van Brummelen EMJ , Cohen RB , Gomez-Roca C , Ejadi S , Stein M , Chan E , Simonelli M , Morosky A , Saraf S , et al. Safety and antitumor activity of the anti-PD-1 antibody pembrolizumab in patients with recurrent carcinoma of the anal canal. Ann Oncol. 2017; 28:1036–1041. 10.1093/annonc/mdx029. 28453692 PMC5406758

[24] McBrien JB , Kumar NA , Silvestri G . Mechanisms of CD8+ T cell-mediated suppression of HIV/SIV replication. Eur J Immunol. 2018; 48:898–914. 10.1002/eji.201747172. 29427516 PMC6531861

